# *De novo* transcriptome analysis in radish (*Raphanus sativus* L.) and identification of critical genes involved in bolting and flowering

**DOI:** 10.1186/s12864-016-2633-2

**Published:** 2016-05-23

**Authors:** Shanshan Nie, Chao Li, Liang Xu, Yan Wang, Danqiong Huang, Everlyne M. Muleke, Xiaochuan Sun, Yang Xie, Liwang Liu

**Affiliations:** National Key Laboratory of Crop Genetics and Germplasm Enhancement, College of Horticulture, Nanjing Agricultural University, Nanjing, 210095 People’s Republic of China; Key Laboratory of Biology and Genetic Improvement of Horticultural Crops (East China) of the Ministry of Agriculture of P.R. China, Nanjing, 210095 People’s Republic of China; Department of Plant Sciences, North Dakota State University, Fargo, ND 58108 USA

**Keywords:** Radish, *De novo* assembly, Bolting and flowering, Transcriptome, Flowering regulatory network

## Abstract

**Background:**

The appropriate timing of bolting and flowering is pivotal for reproductive success in Brassicaceae crops including radish (*Raphanus sativus* L.). Although several flowering regulatory pathways had been described in some plant species, no study on genetic networks of bolting and flowering regulation was performed in radish. In this study, to generate dataset of radish unigene sequences for large-scale gene discovery and functional pathway identification, a cDNA library from mixed radish leaves at different developmental stages was subjected to high-throughput RNA sequencing (RNA-seq).

**Results:**

A total of 54.64 million clean reads and 111,167 contigs representing 53,642 unigenes were obtained from the radish leaf transcriptome. Among these, 50,385 unigenes were successfully annotated by BLAST searching against the public protein databases. Functional classification and annotation indicated that 42,903 and 15,382 unique sequences were assigned to 55 GO terms and 25 COG categories, respectively. KEGG pathway analysis revealed that 25,973 unigenes were classified into 128 functional pathways, among which 24 candidate genes related to plant circadian rhythm were identified. Moreover, 142 potential bolting and flowering-related genes involved in various flowering pathways were identified. In addition, seven critical bolting and flowering-related genes were isolated and profiled by T-A cloning and RT-qPCR analysis. Finally, a schematic network model of bolting and flowering regulation and pathways was put forward in radish.

**Conclusions:**

This study is the first report on systematic identification of bolting and flowering-related genes based on transcriptome sequencing and assembly in radish. These results could provide a foundation for further investigating bolting and flowering regulatory networks in radish, and facilitate dissecting molecular genetic mechanisms underlying bolting and flowering in Brassicaceae vegetable crops.

**Electronic supplementary material:**

The online version of this article (doi:10.1186/s12864-016-2633-2) contains supplementary material, which is available to authorized users.

## Background

The formation of bolting and flowering, marking the developmental transition from vegetative growth to flowering, is one of the most important developmental traits in plant life cycle. Appropriate bolting and flowering time is crucial to ensure reproductive success and high agricultural productivity for crop plants [1, 2]. In *Arabidopsis thaliana*, some molecular and genetic studies have revealed that a complex gene-network has evolved to orchestrate the initiation of bolting and flowering and is regulated by diverse environmental and endogenous factors such as temperature, light signals, day length, developmental stage and plant hormones [3–5].

Considerable studies in *Arabidopsis* revealed that a set of genes involved in flowering control have been discovered and characterized and integrated into the genetic circuitry of flowering and multiple flowering pathways, including pathways of photoperiod, vernalization, aging, autonomous and gibberellin (GA) [2, 3, 6]. The transition from vegetative growth to reproductive development is regulated by the interplays of central flowering genes involved in different flowering pathways [7, 8]. The pathways of autonomous and vernalization are integrated by *FLOWERING LOCUS C* (*FLC*), which is a main flowering repressor and encodes a MADS-box transcription factor [9]. The expression level of *FLC* is correlated with plant flowering time and overexpression of *FLC* causes late flowering [2, 9]. In addition, *SUPPRESSOR OF OVEREXPRESSION OF CONSTANS 1* (*SOC1*), *FLOWERING LOCUS T* (*FT*) and *LEAFY* (*LFY*) are showed to be convergence points for different flowering pathways and defined as flowering pathway integrators [7, 8, 10]. In the past decade, a number of functional genes and regulatory pathways related to flowering time had also been discovered in many crops including rice [11, 12], maize [13], strawberry [14] and soybean [15].

With the development of next-generation sequencing (NGS) technologies, several platforms such as Illumina Solexa, Roche 454 and ABI SOLiD, have been proven to be powerful and efficient tools for advanced genomic researches, including the whole genome re-sequencing and transcriptome sequencing [16, 17]. Recently, the advent of NGS-based RNA sequencing (RNA-seq) of transcriptome has provided an opportunity for large-scale discovery and profiling of functional genes involved in diverse biological processes [18, 19], such as floral scent biosynthesis in wintersweet [20], discovery of disease-resistance genes (NBS-LRR genes) in *Camelina sativa* [21], biosynthesis and accumulation of lignin in celery [22] and terpenoid biosynthesis in pitanga [23]. RNA-seq has also been used for the discovery of genes related to flowering time regulation and flower development in some plant species including bamboo [24, 25], *Eichhornia paniculata* [26], *Lagerstroemia indica* [27], *Ipomoea nil* [28] and sweet potato [29]. However, genome-wide identification of flowering-related functional genes is still lacking in root vegetable crops.

Radish (2*n* = 2*x* = 18) is an important annual or biennial root vegetable crop of Brassicaceae family and belongs to LD (long-day) plant. Bolting and flowering are integral phases in the complete life cycle of radish. Premature bolting is a destructive problem that limits vegetative growth and reduces yield and quality of economic products in Brassicaceae crops, especially the radish grown in spring. The timing of bolting and flowering is of great importance for high productivity. Recently, the draft genomic sequences of radish have been assembled [30], providing useful database for genomic research in radish. Moreover, some recent studies concerning the radish transcriptome assembly and analysis were reported, and a list of radish unique sequences were generated from various radish tissues [31, 32]. Previous studies reported that several flowering genes have been isolated and characterized by expression profiling and transgenic approaches [33–35]. However, the genetic networks of bolting and flowering regulation in radish are poorly understood. To characterize the radish leaf transcriptome and identify critical genes involved in bolting and flowering of radish, transcript sequences from radish leaves during different developmental phases were isolated and sequenced by Illumina sequencing method. The goal of this study was to obtain a complete set of assembled unigenes and transcripts in radish leaves, and to identify transition of bolting and flowering related genes involved in flowering-time regulatory networks. The full-length cDNA sequences of seven flowering related genes were isolated by T-A cloning and further compared with the assembled transcript sequences from radish leaf transcriptome. Moreover, expression patterns of seven isolated genes were validated by RT-qPCR analysis. Furthermore, a graphical network of bolting and flowering-time regulation was proposed. These findings could provide a solid foundation for better understanding of bolting and flowering-time regulatory networks in radish, and provide significant insights into molecular genetic mechanisms underlying bolting and flowering regulation in Brassicaceae vegetable crops.

## Results and discussion

### Illumina sequencing and *de novo* transcriptome assembly

**Fig. 1:**
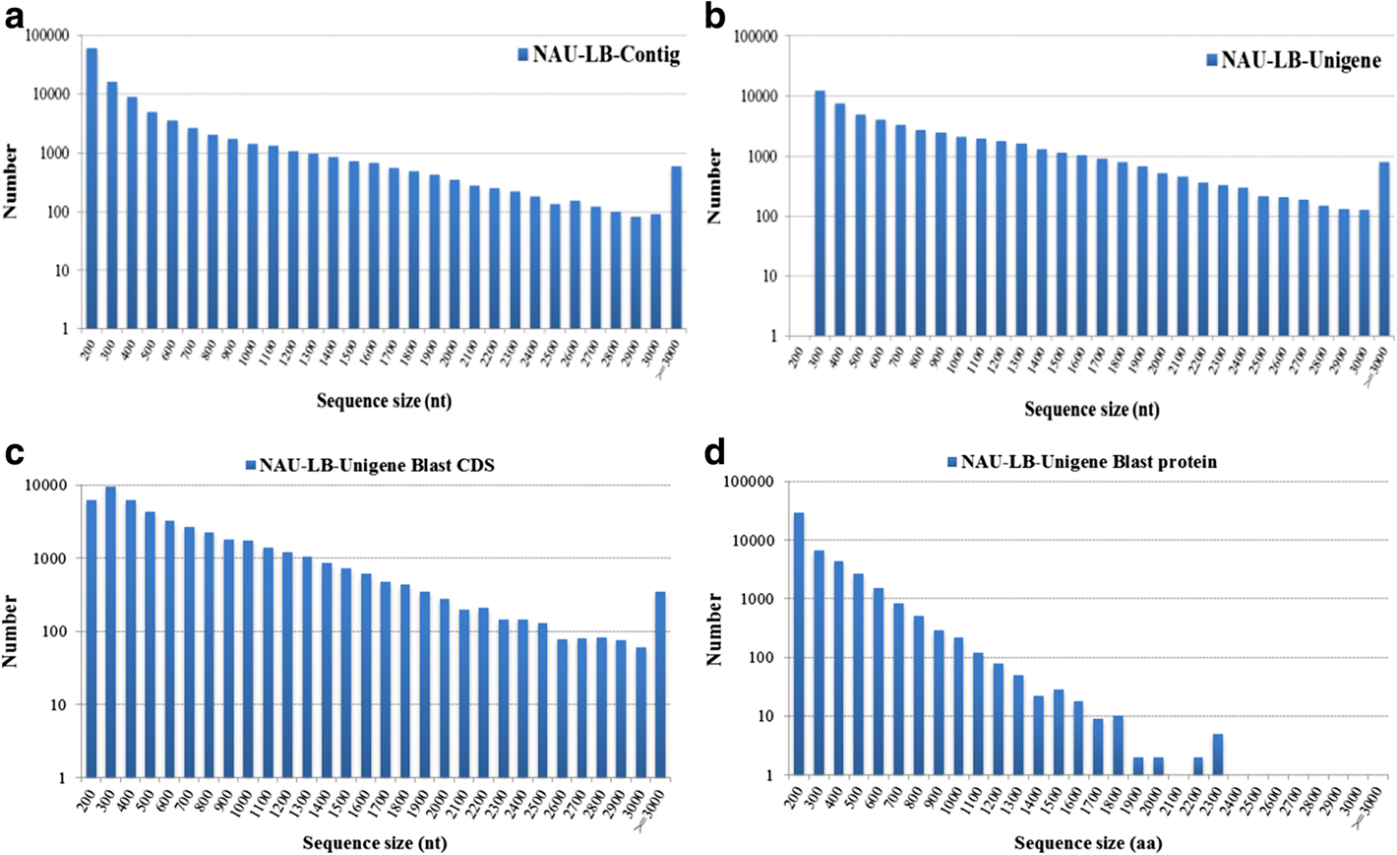
Overview of the radish leaf transcriptome assembly. The length distribution of assembled contigs (**a**) and unigenes (**b**), and the CDS (**c**) and predicted proteins (**d**) by BLASTx alignment in radish

RNA-seq is a useful approach for obtaining a complete set of transcripts from certain plant tissue at specific developmental stage or under certain physiological condition [18, 19]. To obtain a comprehensive overview of radish leaf transcriptome, a cDNA library from radish leaves of late-bolting ‘NAU-LU127’ at different developmental stages was constructed and sequenced using Illumina RNA-seq. A total of 58,602,240 pair-end reads were generated from the radish leaf transcriptome, named ‘NAU-LB’. After the removal of adapter sequences, ambiguous reads and low quality reads, 54,637,700 high-quality clean reads comprising of 4,917,393,000 nucleotides (nt) were obtained with an average length of 90 nt and average GC content of 47.12 % (Table 1). All clean reads were assembled into 111,167 contigs using the Trinity program [36], with a mean length of 429 nt and an N50 length of 476 nt (Table 2). Then, these contigs were assembled into 53,642 unigenes with a total length of 43,022,520 nt, an average length of 802 nt and an N50 length of 1169 nt (Table 2). Length distributions of assembled contigs and unigenes were analyzed and demonstrated in Fig. 1.

**Table 1:** Summary of Illumina transcriptome sequencing from radish leaves

Samples	NAU-LB
Total raw reads	58,602,240
Total clean reads	54,637,700
Total clean nucleotides (nt)	4,917,393,000
Average length of clean reads (nt)	90
Q20 percentage	98.47 %
N percentage	0.01 %
GC percentage	47.12 %

**Table 2:** Summary of radish leaf transcriptome assembly

	Contig	Unigene
Total number	111,167	53,642
Total Length(nt)	47,730,600	43,022,520
Mean Length(nt)	429	802
N50	476	1169
Total consensus sequences	–	53,642
Distinct clusters	–	22,665
Distinct singletons	–	30,977

Using the RNA-seq platform and Trinity program, a number of assembled unigenes were also generated from radish leaves and roots [31, 32]. Zhang et al. [31] reported that 28,410 unigenes with an average length of 394 bp and an N50 of 422 bp was generated from radish leaves by transcriptome assembly. Wang et al. [32] showed that 73,084 unigenes with a mean length of 763 nt and an N50 of 1095 nt were obtained from radish root transcriptome. To evaluate the quality of assembly results and transcriptome data, comparison analysis showed that the average length and N50 length of unigenes obtained in this study were longer than that in previous studies on radish, indicating these sequencing data were of high quality and sufficient quantity for further analysis.

To estimate the expression levels of assembled unigenes in radish leaf transcriptome, the FPKM (Fragments Per kb per Million fragments) method [37] was used for the calculation of unigene expression level. The global detailed information of all the assembled unigenes was showed in Additional file 1. It is well known that the *de novo* transcriptome sequencing had been successfully applied for many vegetable crops including *B. oleracea* [38], *B. napus* [39] and celery [22]. Consistent with these studies, the transcript sequences from radish leaf transcriptome could provide a valuable resource for comprehensively identifying specific developmental processes, pathways and functional genes in radish.

### Functional annotation of radish leaf transcriptome

To assign putative gene functions and annotations of all the assembled unigenes, BLASTx alignment was performed with an *E*-value threshold of 1.0E-05 against the public protein databases including NCBI non-redundant protein (NR), Swiss-Prot, Clusters of Orthologous Groups (COG) and Kyoto Encyclopedia of Genes and Genomes (KEGG). A total of 50,385 unigenes (93.93 % of all unigenes) were annotated and matched to one or more of the public protein databases (Table 3; Additional file 1). The best aligning results were used to decide the sequence direction of each assembled unigene. The size distributions for the coding sequence (CDS) and predicted proteins were analyzed and shown in Fig. 1. Nevertheless, a small proportion of sequences (3,257 unigenes, 6.07 %) was unannotated and showed no matches to any above protein databases, which might represent radish-specific transcriptomic sequences in radish leaves.

**Table 3:** The annotations of radish leaf unigenes against the public databases

Sequence file	NAU-LB-Unigene	Percent (%)
NR	46,660	86.98
NT	49,051	91.44
Swiss-Prot	30,955	57.71
KEGG	25,973	48.42
COG	15,382	28.68
GO	42,903	79.98
ALL	50,385	93.93

**Fig. 2:**
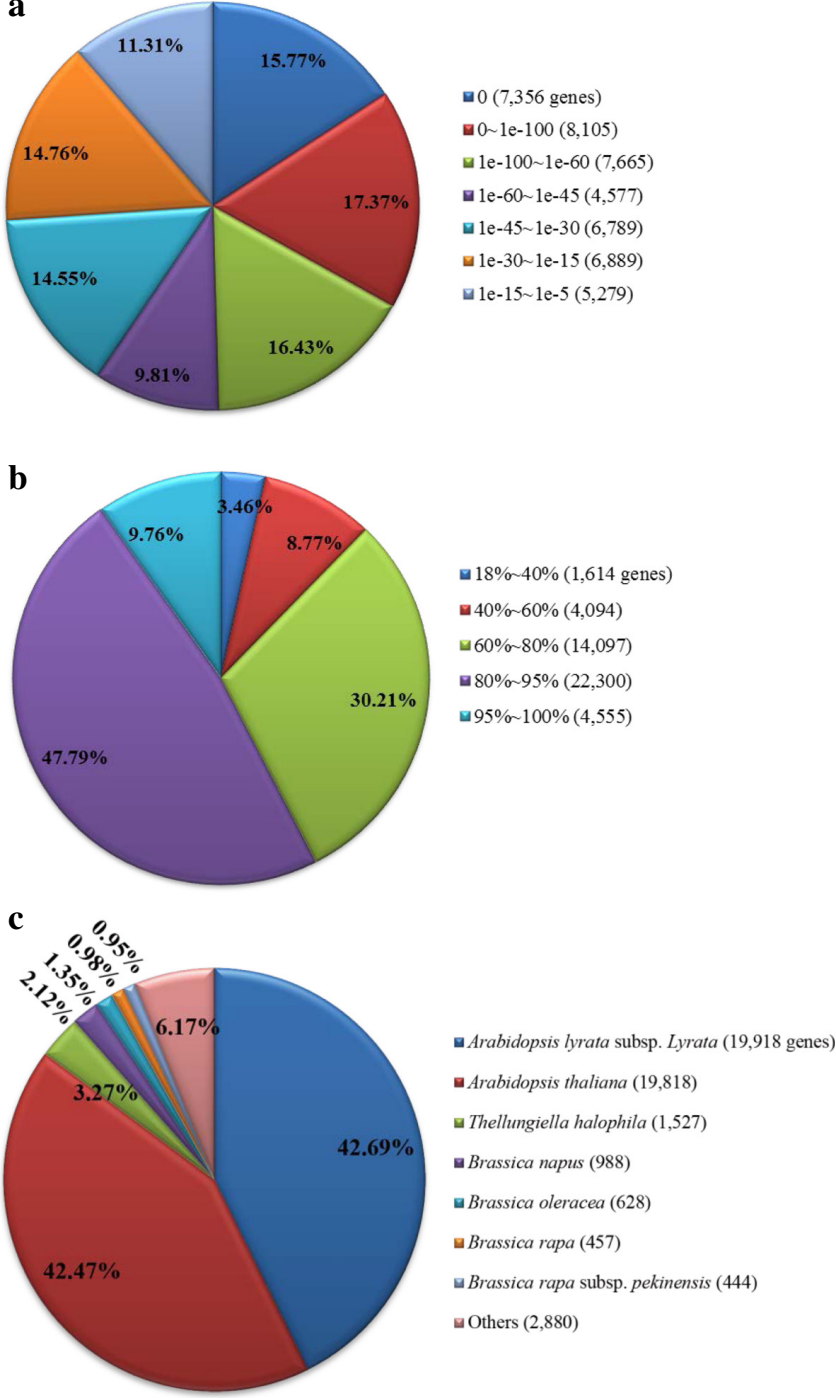
Characteristics of sequence homology of radish unigenes against NR database. **a** *E*-value distribution of BLAST hits for each unigene with an *E*-value cutoff of 1.0E-05. **b** Similarity distribution of the top BLAST hits for each unigene. **c** Species distribution of the top BLAST hits

By sequence similarity searching against the NCBI NR database, 46,660 annotated sequences (86.98 % of all unigenes) were detected and showed significant similarities to known proteins (Table 3). According to the *E*-value distribution of significant hits against the NR database, 59.38 % of the matched sequences showed high homologies with the *E*-value < 1.0E-45, while 40.62 % of the top hits had *E*-value in the range of 1.0E-05 to 1.0E-45 (Fig. 2a). The similarity distribution of the top BLAST hits revealed that 57.55 % of the matched sequences were in similarity higher than 80 % (Fig. 2b). Further analysis of homologies among different plant species showed that 42.69 % of the annotated sequences matched to sequences from *Arabidopsis lyrata*, followed by *A. thaliana* (42.47 %), *Thellungiella halophila* (3.27 %) and *B. napus* (2.12 %) (Fig. 2c). The species distribution revealed that most of annotated unigenes had high hits with sequences from the Brassicaceae species, suggesting that the assembly and annotation of radish leaf transcriptome are correct and reliable.

### GO and COG classification

**Fig. 3:**
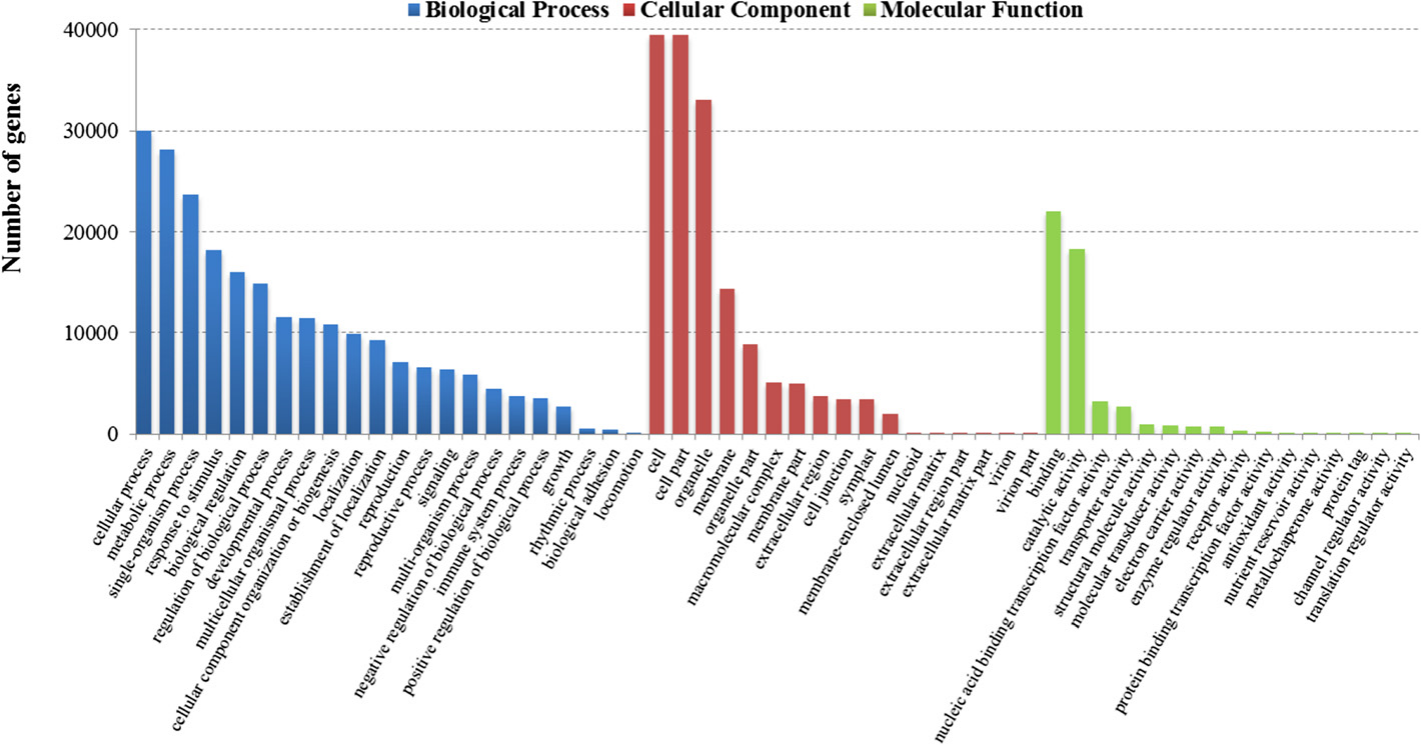
GO classification of the radish leaf transcriptome

Based on the BLASTx results against the NR database, Blast2GO program was performed to obtain Gene Ontology (GO) functional annotations and categorizations of these assembled unigenes. As a result, 42,903 unique sequences were annotated and classified into 55 GO classes including 22 biological processes, 17 cellular components and 16 molecular functions (Fig. 3). Under the biological process category, ‘cellular process’ (30,031 sequences), ‘metabolic process’ (28,182) and ‘single-organism process’ (23,665) were prominently represented. Within these cellular components, ‘cell’ (39,426), ‘cell part’ (39,426) and ‘organelle’ (33,017) were the most highly represented categories. For the molecular function category, the largest proportion of genes were clustered into ‘binding’ (22,010), ‘catalytic activity’ (18,298) and ‘nucleic acid binding transcription factor activity’ (3,242). Additionally, only a few sequences were assigned to ‘virion’, ‘virion part’, ‘channel regulator activity’ and ‘translation regulator activity’ terms with less than ten sequences.

**Fig. 4:**
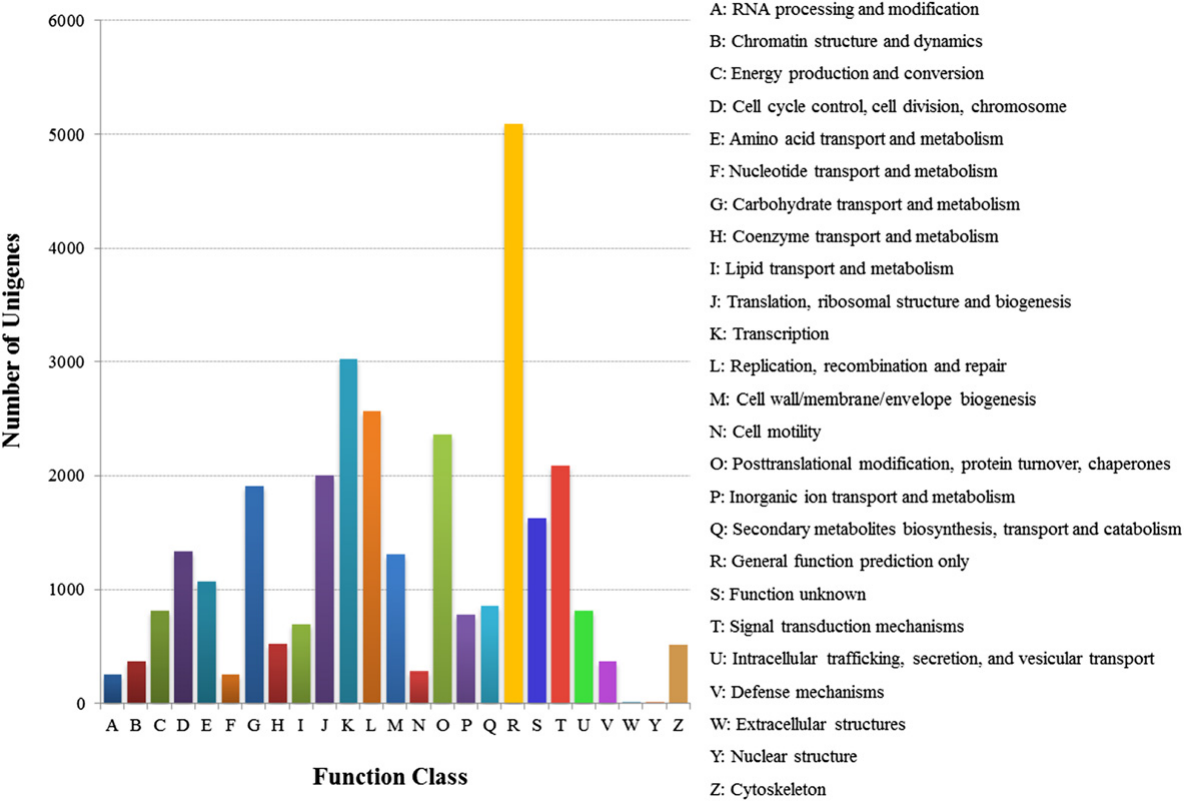
COG functional classification of the radish leaf transcriptome

To further predict and classify the possible functions of radish unigenes, these unique sequences from radish leaf transcriptome were aligned to the COG database. The results showed that 15,382 unigenes (28.68 % of all unigenes) were assigned to 25 COG categories (Table 3; Fig. 4). Of these, the largest category was ‘General function prediction only’ (5,088 sequences), followed by ‘Transcription’ (3,019), ‘Replication, recombination and repair’ (2,568), ‘Posttranslational modification, protein turnover, chaperones’ (2,362) and ‘Signal transduction mechanisms’ (2,091); whereas the clusters of ‘Extracellular structures’ (3) and ‘Nuclear structure’ (7) represented the smallest classifications. These assigned functions of unigenes covered a wide range of GO and COG classifications, indicating that the transcriptome data from radish leaves represented a broad variety of transcripts in radish.

### Identification of functional genes involved in circadian rhythm

To systematically study the complex biological functions of genes and identify active biological pathways in radish leaves, the assembled unigenes were mapped against the KEGG database using BLASTx analysis with a cut-off *E*-value of 1.0E-05. In total, 25,973 unigenes (48.42 % of all unigenes) had significant matches to the database and were assigned to 128 KEGG pathways (Table 3; Additional file 2). The most dominant pathways were ‘metabolic pathways’ (ko01100; 5,522 unigenes), ‘biosynthesis of secondary metabolites’ (ko01110; 2,573), ‘plant hormone signal transduction’ (ko04075; 1,495), ‘plant-pathogen interaction’ (ko04626; 1,436) and ‘RNA transport’ (ko03013; 1,088) (Additional file 2). The results were comparable with the previous transcriptome profiles in radish root, in which 33,567 unigenes were clustered into 128 KEGG pathways [32].

**Fig. 5:**
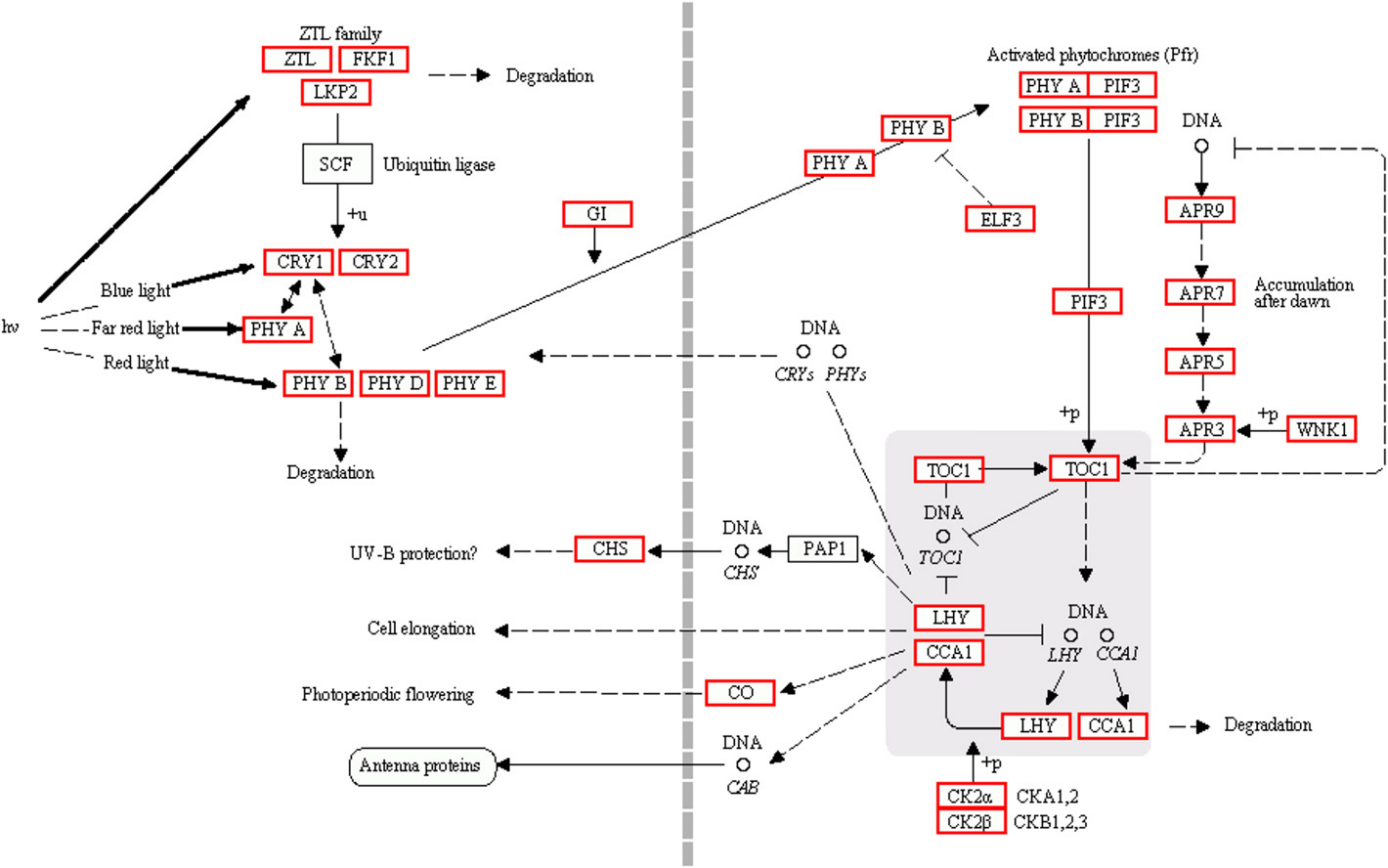
The pathway of circadian rhythm in radish. The circadian related genes identified in radish are in *red. Arrows* indicate positive regulation and *bars* indicate negative regulation

Significantly, pathway-based analysis revealed that a total of 249 transcript sequences representing 24 rhythm related candidate genes were identified for the ‘circadian rhythm-plant’ pathway (ko04712) (Fig. 5; Additional file 3). These rhythm related genes including *CONSTANS* (*CO*, K12135), *CIRCADIAN CLOCK ASSOCIATED 1* (*CCA1*, K12134), *EARLY FLOWERING 3* (*ELF3*, K12125), *LATE ELONGATED HYPOCOTYL* (*LHY*, K12133) and *GIGANTEA* (*GI*, K12124), were involved in many rhythmic processes including photoperiodic flowering, UV-B protection and cell elongation (Fig. 5). In addition, several identified potential genes including *LOV KELCH PROTEIN 2* (*LKP2*, K12117), *CRYPTOCHROME 1* (*CRY1*, K12118), *CRYPTOCHROME 2* (*CRY2*, K12119), *PHYTOCHROME A* (*PHYA*, K12120), *PHYTOCHROME B* (*PHYB*, K12121), *PHYTOCHROME D* (*PHYD*, K12122) and *PHYTOCHROME E* (*PHYE*, K12123), were implicated in plant light signaling pathways [40, 41].

Plant circadian clock as an internal timekeeper controls daily and seasonal changes of many rhythmic processes according to the day length and photoperiod [42]. Photoperiodic flowering control is linked to circadian clock by the transcriptional expression of *CO* which modulates plant flowering time and initiates floral development [41, 43–45]. *CO* encoding a zinc finger protein is central component of photoperiodic flowering pathway and could integrate endogenous and environmental information to trigger plant flowering at proper time [43, 46]. It was reported that the expression of *CO* was regulated by the circadian clock and the peak expression occurred at the end of the day under long day conditions [41, 43, 46]. In the present study, 46 transcripts were annotated and identified to be homologous sequences of *CO* (Additional file 3). Understanding the roles of circadian-regulated genes responsible for controlling flowering could contribute to study the interplays of photoperiod and circadian clock.

### Identification of functional genes involved in flowering pathways in radish

Functional genetic studies have evidenced that several coordinate flowering pathways form a complex genetic network and control the development transition of flowering [1, 3]. More than 200 flowering-related genes implicated in the complexity of flowering have been identified and characterized in model crop *Arabidopsis* [2, 3]. In this study, to comprehensively identify the candidate genes putatively implicated in bolting and flowering regulation in radish, a local BLASTx similarity search was performed against *A. thaliana* flowering genes which were downloaded from NCBI database. A total of 142 potential bolting and flowering-related genes including 373 transcript sequences were identified and shared higher similarity to the homologous genes from *Arabidopsis* (Additional files 4 and 5). According to the reported flowering pathways and regulatory networks, these identified bolting and flowering-related genes were characterized and involved in various flowering pathways (Table 4), including photoperiod/circadian clock, vernalization, autonomous, GA and aging pathways [2, 3]. The BLAST searching showed that 71 potential functional genes including *CONSTANS-LIKE 1* (*COL1*), *TEMPRANILLO 1* (*TEM1*), *TEM2*, *TERMINAL FLOWER 2* (*TFL2*) and *FLOWERING LOCUS D* (*FD*), were identified and implicated in photoperiod pathway (Table 4; Additional file 4). In addition, the pathways of vernalization, autonomous, GA and aging contained 28, 8, 16 and 4 candidate genes, respectively. These results revealed that the known genetic flowering pathways and many critical flowering genes shared a high degree of conservation in radish and *Arabidopsis*.

**Table 4:** The identified candidate genes involved in various flowering pathways of radish

Gene name	Number of unigenes	Flowering pathway
*ABH1/ENS*	1	Photoperiod
*AGL12*	1	Others
*AGL14*	1	Others
*AGL15*	2	Photoperiod
*AGL16*	1	Others
*AGL18*	3	Photoperiod
*AGL19*	2	Vernalization
*AGL24*	2	Photoperiod
*AGL6*	7	Photoperiod
*AGL8/FUL*	4	Photoperiod
*AP1*	5	Photoperiod
*AP2*	6	Photoperiod
*AP3*	3	Photoperiod
*ARP4*	3	Photoperiod
*ARP6*	1	Vernalization
*ARR3*	2	Photoperiod
*ATH1*	3	flower development
*ATX1*	6	flower development
*CBF1*	1	Vernalization
*CBF2*	1	Vernalization
*CCA1*	3	Photoperiod/Circadian clock
*CCR2*	3	Autonomous
*CDF1*	5	Photoperiod
*CDF2*	1	Photoperiod
*CDF3*	1	Photoperiod
*CO*	3	Photoperiod/Circadian clock
*COL1*	2	Photoperiod/Circadian clock
*COL2*	1	Photoperiod/Circadian clock
*COL3*	1	Photoperiod
*COL4*	3	Photoperiod
*COL5*	4	Photoperiod
*COL9*	2	Photoperiod/Circadian clock
*COP1*	1	Photoperiod
*CRY1*	4	Photoperiod/Circadian clock
*CRY2*	1	Photoperiod/Circadian clock
*CSTF64*	2	Vernalization
*CSTF77*	4	Vernalization
*DDF1*	7	GA
*DDF2*	2	GA
*DDL*	1	flower development
*EBS*	3	Photoperiod
*EFS*	2	flower development
*ELF3*	6	Photoperiod/Circadian clock
*ELF4*	1	Photoperiod/Circadian clock
*ELF5*	1	Photoperiod
*ELF6*	1	Photoperiod
*ELF7*	2	Photoperiod
*ELF8*	1	Photoperiod
*ELF9*	1	Photoperiod
*EMF1*	1	Vernalization
*ESD4*	1	Vernalization
*FCA*	2	Autonomous
*FD*	4	Photoperiod
*FES1*	2	Vernalization
*FIE*	3	others
*FIP1*	2	Vernalization
*FIP2*	1	Vernalization
*FLC*	3	integrator
*FLD*	1	Autonomous
*FLK*	4	Autonomous
*FLM/MAF1*	1	Photoperiod
*FLP1*	1	flower development
*FPA*	3	Autonomous
*FPF1*	1	GA
*FRI*	1	Vernalization
*FT*	1	integrator
*FVE/MSI4*	7	Autonomous
*FY*	1	Autonomous
*GA3*	1	GA
*GAI*	2	GA
*GI*	5	Photoperiod/Circadian clock
*GID1A*	2	GA
*GID1B*	4	GA
*GID1C*	2	GA
*GNC*	2	GA
*GNL*	2	GA
*HUA2*	5	Vernalization
*HYL1/DRB1*	2	others
*LCL1*	3	Photoperiod/Circadian clock
*LD*	4	Autonomous
*LDL2*	1	flower development
*LFY*	3	integrator
*LHP1*	3	Vernalization
*LHY*	2	Photoperiod/Circadian clock
*LKP2*	3	Photoperiod/Circadian clock
*LWD1/ATAN11*	1	Photoperiod/Circadian clock
*LWD2*	1	Photoperiod/Circadian clock
*MBD9*	6	Photoperiod
*MYB33*	2	GA
*MYB65*	3	GA
*NF-YA1/HAP2A*	1	Photoperiod
*NF-YA4*	3	Photoperiod
*NF-YB1*	2	Photoperiod
*NF-YB2*	3	Photoperiod
*pEARLI*	2	Vernalization
*PHYA*	2	Photoperiod/Circadian clock
*PHYB*	2	Photoperiod/Circadian clock
*PHYC*	3	Photoperiod/Circadian clock
*PHYD*	1	Photoperiod/Circadian clock
*PHYE*	9	Photoperiod/Circadian clock
*PI*	2	flower development
*PIE1*	4	Photoperiod
*PRC2/CLF/EMF2*	5	Vernalization
*PRR3*	3	Photoperiod/Circadian clock
*PRR7*	7	Photoperiod/Circadian clock
*PRR9*	1	Photoperiod/Circadian clock
*REF6*	6	Vernalization
*RGA*	2	GA
*RGL1*	2	GA
*RGL2*	1	GA
*SDG10/SWN*	2	Vernalization
*SEP1/AGL2*	4	Others
*SEP2/AGL4*	4	Others
*SEP3/AGL9*	6	flower development
*SHP1/AGL1*	3	flower development
*SHP2/AGL5*	3	flower development
*SKB1*	2	Vernalization
*SOC1/AGL20*	2	integrator
*SPA*	9	Photoperiod
*SPL*	2	Age
*SPL3*	4	Age
*SPL5*	2	Age
*SPL9*	4	Age
*SPY*	3	GA
*SUF4*	8	Vernalization
*SVP/AGL22*	3	Vernalization
*TEM1*	6	Photoperiod
*TEM2*	7	Photoperiod
*TFL2*	3	Photoperiod
*TIC*	10	Photoperiod/Circadian clock
*TOE1*	3	Photoperiod
*TOE2*	2	Photoperiod
*TOE3*	3	Photoperiod
*TPS1*	4	Others
*VEL1*	2	Photoperiod
*VIN3*	1	Vernalization
*VIP1*	2	Vernalization
*VIP2*	2	Vernalization
*VRN1*	9	Vernalization
*VRN2*	2	Vernalization
*WNK1*	3	Photoperiod/Circadian clock

**Fig. 6:**
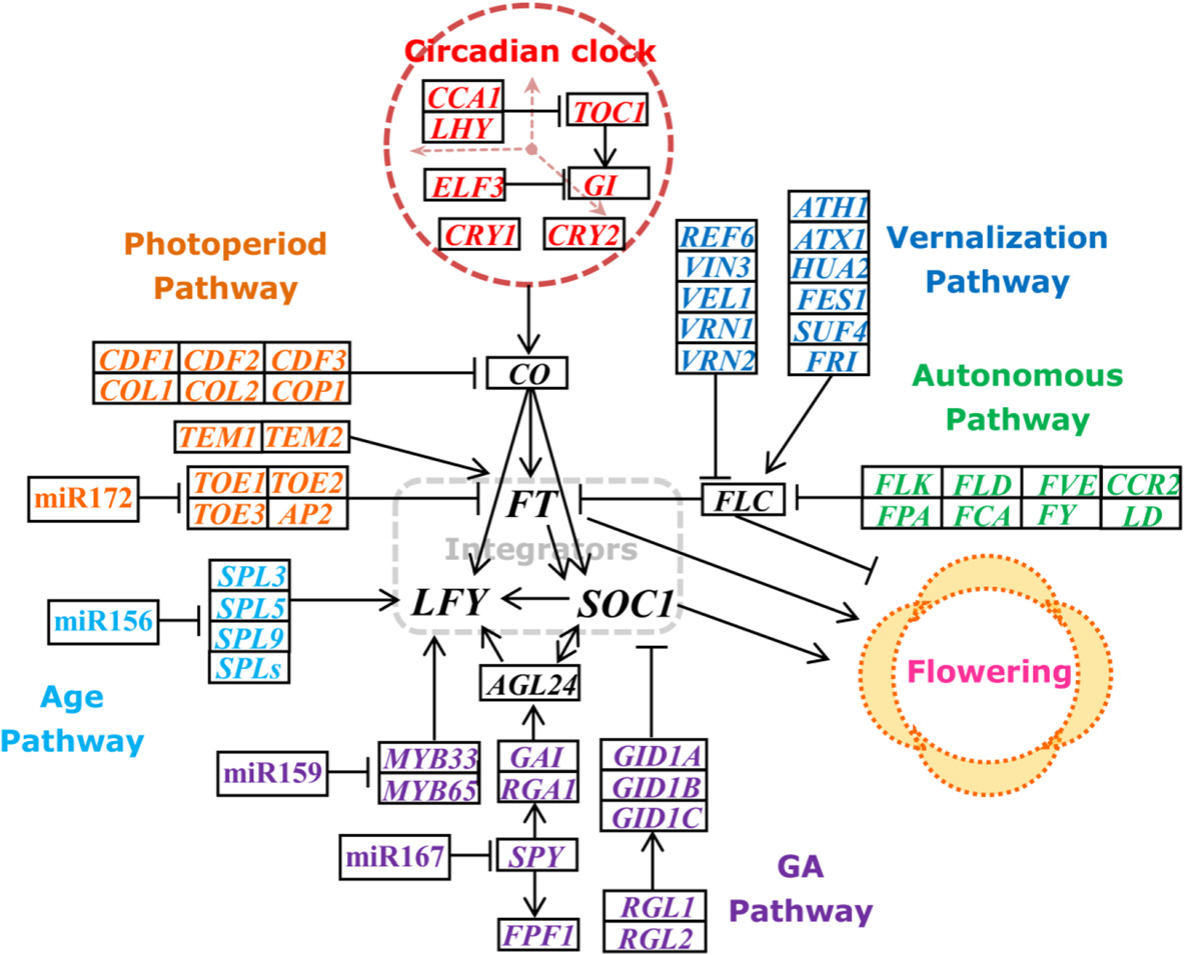
The putative schematic network of bolting and flowering regulation in radish. The genes in *black* were the flowering pathway integrators. The miR156, miR172, miR159 and miR167 were identified in our previous study [35]. *Arrows* indicate positive regulation and *bars* indicate negative regulation

Moreover, to summarize and characterize the identified functional genes and different flowering pathways, a putative schematic network of bolting and flowering regulation in radish was proposed (Fig. 6). More importantly, many key players of flowering regulation including *SOC1*, *CO*, *FT* and *FLC* and several functional genes associated with floral meristem identity and flower development including *APETALA1* (*AP1*), *SEPALLATA1-3 (SEP1-3), AGAMOUS-LIKE 24* (*AGL24*) and *LFY*, were identified in this study (Table 4; Fig. 6). Similar results were also found in previous genetic analysis studies on rice [12], barley [47], soybean [15], maize [13] and strawberry [14]. These results provide essential information for functional analysis of bolting and flowering genes and regulatory pathways, and facilitate further genetically investigating in radish.

### Characterization of bolting and flowering genes and regulatory pathways in radish

Studies on developmental switch of *Arabidopsis* flowering have demonstrated that multiple different flowering pathways converge at several flowering pathway integrators including *FT*, *SOC1* and *LFY* to determine the flowering time [2, 7, 10]. These integrators are regulated by two key upstream genes *CO* and *FLC*, which antagonistically control flowering [1, 7]. *FLC* as a main flowering repressor integrates autonomous and vernalization pathways and negatively regulates the expressions of *FT* and *SOC1* [48, 49]. Previous studies had identified the homologous genes of *FLC* in *B. rapa* [50], sweet potato [29] and orange [51]. In this study, three unique sequences of *FLC* gene, CL1584.Contig1, CL1584.Contig2 and CL1584.Contig3, were identified and matched to the homologs of *AtFLC* (Additional file 4). After undergoing vernalization process, a long exposure to low temperature, the plants permit to initiate the phase transition of flowering. While the process could be repressed by the high level of *FLC* [9, 52], which requires its activator *FRIGIDA* (*FRI*), a plant-specific gene conferring vernalization response in *Arabidopsis* [53]. In addition, genomic analysis suggested that many genes were involved in vernalization pathway and played roles in flowering control [2, 15]. As expected, the vernalization-response genes including *FRI*, *FRIGIDA ESSENTIAL 1* (*FES1*), *VERNALIZATION INSENSTIVE 3* (*VIN3*), *VERNALIZATION 1* (*VRN1*) and *VRN2*, were also identified in this study (Fig. 6; Table 4), which was consistent with previous studies on flowering gene discovery [15, 24, 25, 51].

Additionally, we also found transcript sequences matching to the candidate genes of *SOC1* (CL4258.Contig1 and CL4258.Contig2), *AGL24* (CL2892.Contig1 and CL2892.Contig2) and *LFY* (Unigene29702, Unigene30559 and Unigene28363) (Additional file 4). Both *SOC1* and *AGL24* belonged to MADS-box genes, and their interaction directly regulated the expression of *LFY* and determined the flowering time [10, 49, 54, 55]. The family of MADS-box transcription factor is a major group of regulators controlling floral transition in *Arabidopsis* and act in floral organ specification and floral development in flowering plants [56–58]. Our studies also identified some members of MADS family such as *AGLs*, *AP1*, *AP2*, *SHORT VEGETATIVE PHASE* (*SVP*) and *FLOWERING LOCUS M* (*FLM*), which might participate in the development and flowering regulation of radish (Fig. 6).

Moreover, radish transcripts which showed to be homologous genes in GA pathway included *FLOWERING PROMOTING FACTOR 1* (*FPF1*), *GIBBERELLIC ACID INSENSITIVE* (*GAI*), *MYB33*, *MYB65* and *SPINDLY* (*SPY*) (Table 4). It was reported that *MYB33* and *MYB65* were the target genes of miR159 which could control the flowering time *via* the GA pathway [59, 60], while *SPY* targeted by miR167 was a negative regulator of gibberellin signaling and functioned in mediating flowering time [61]. Furthermore, some bolting and flowering-related critical miRNAs including miR156, miR172, miR159 and miR167 were also identified in our previous study [35] (Fig. 6). These findings revealed that the transcriptome dataset of radish leaves could be used for the discovery and profiling of functional genes involved in various developmental pathways and processes in root vegetable crops.

### Isolation and validation of bolting and flowering-related genes

Based on the annotation analysis and BLAST search of radish leaf transcriptomic sequences, the full-length sequences of functional genes related to flowering regulation were obtained by transcript sequence assembly. To evaluate the quality of assembled sequences and annotation results from the NGS sequencing, the sequences of several flowering genes from T-A cloning and transcript assembly were subjected to comparative analysis. The full-length cDNA sequences of seven randomly selected bolting and flowering-related genes including *AGL24*, *AP2*, *FPF1*, *LUMINIDEPENDENS* (*LD*), *VRN2*, *SOC1* and *SVP*, were isolated from radish late-bolting line ‘NAU-LU127’ using specific primers and sequenced by the Sanger methods (Table 5). Comparative analysis showed that the full length of these isolated gene sequences ranged from 365 bp to 2,504 bp, and all of them contained the complete open reading frame (ORF) (Table 5). These assembled transcripts had more than 92 % coverage to the corresponding full-length genes, with more than 91 % similarity of ORF. These results revealed that the transcript sequences from radish leaf transcriptome could be used for the identification and isolation of more functional genes related to bolting and flowering in radish.

**Table 5:** Sequence analysis of the isolated genes related to bolting and flowering in radish

Gene name	Full length cDNA (bp)	Coverage (%)	ORF length (bp)	ORF similarity (%)
*RsAGL24*	828	99.28	666	99.85
*RsAP2*	1334	94.15	1302	94.01
*RsFPF1*	365	98.18	330	98.18
*RsLD*	2504	99.40	2448	99.58
*RsSOC1*	724	100	642	100
*RsSVP*	801	92.52	726	91.74
*RsVRN2*	1342	99.93	1281	99.92

**Fig. 7:**
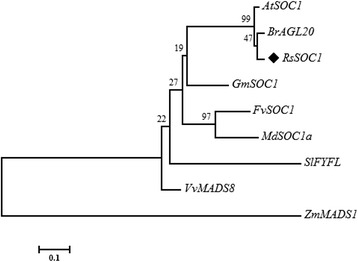
The phylogenetic analysis of *RsSOC1* and the homologous genes in other plants using MEGA 6.0 software with Neighbor-Joining algorithm. The abbreviations for the respective species names are as follows: At, *Arabidopsis thaliana*; Br, *Brassica rapa*; Gm, *Glycine max*; Fv, *Fragaria vesca*; Md, *Malus domestica*; Sl, *Solanum lycopersicum*; Vv, *Vitis vinifera*; Zm, *Zea mays*

Remarkably, the assembled transcript sequence of *SOC1* had 100 % coverage and 100 % ORF similarity to that from T-A cloning (Table 5). The role and function of *SOC1* is mainly promoting flowering and floral meristem identity, and the photoperiod, vernalization and GA pathways converge on *SOC1* to control the flowering time [10]. Phylogenetic relationship between *RsSOC1* and the homologous genes in other plants was analyzed using MEGA 6.0 software (Fig. 7). The phylogenetic analysis indicated that the *SOC1* homologs were clustered into two subgroups, and *RsSOC1* shared the closest relationship with the *BrAGL20* from *B. rapa* and was clearly separated from *ZmMADS1*, a *ZmSOC1-*like gene from *Zea mays*.

### Expression profiles by RT-qPCR

**Fig. 8:**
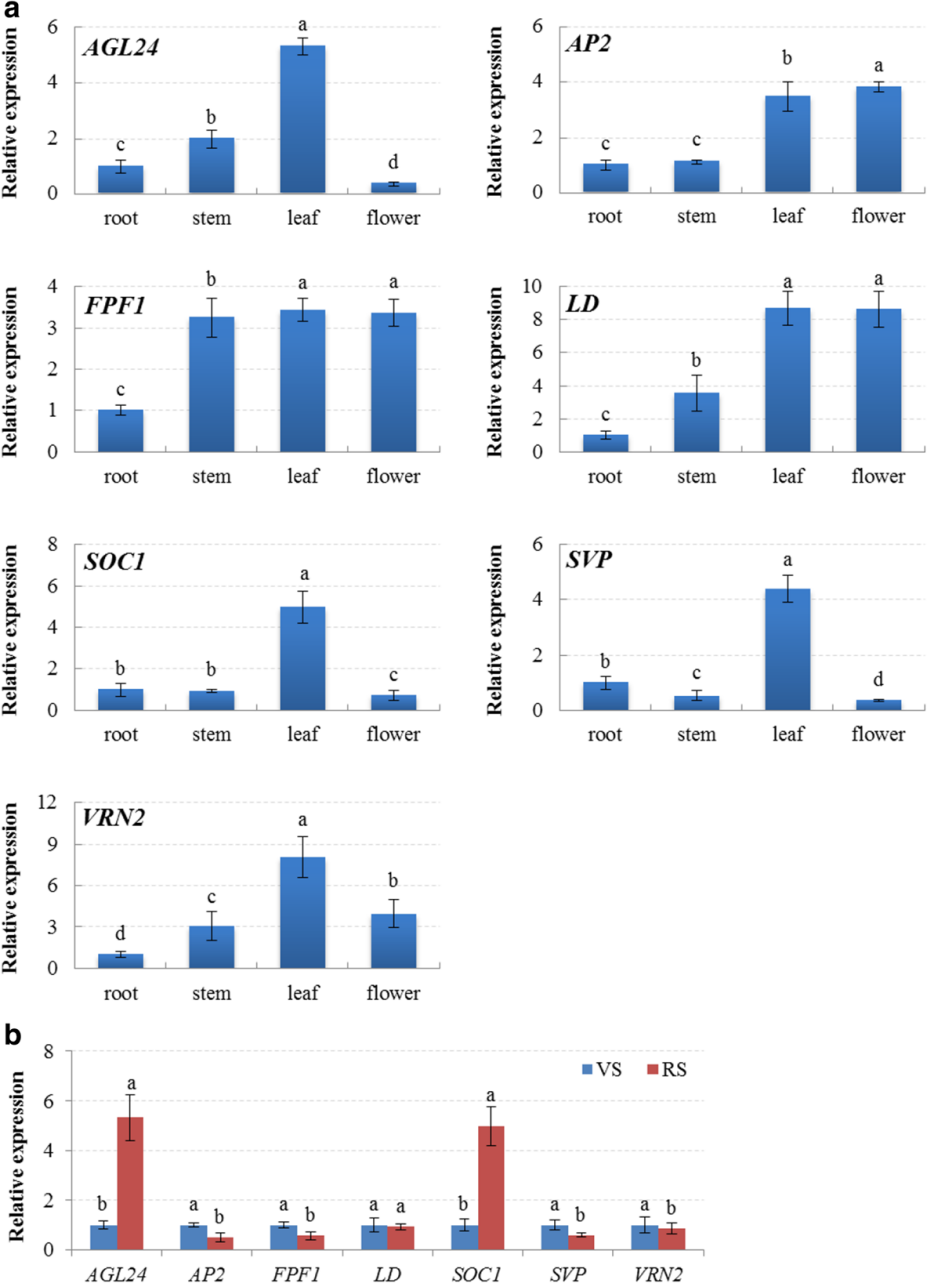
The spatial-temporal expression profiling of bolting and flowering-related genes by RT-qPCR analysis. **a** The expression patterns of seven genes in different radish tissues. **b** The expression patterns of seven genes at vegetative stage (VS) and reproductive stage (RS). Each *bar* shows the mean ± SE of triplicate assays. The values with *different letters* indicate significant differences at *P* < 0.05 according to Duncan’s multiple range tests

To investigate the spatial-temporal expression patterns of bolting and flowering-related genes, the relative expression levels of seven isolated candidate genes in various tissues and at different developmental stages were validated by RT-qPCR (Reverse transcription quantitative real-time PCR) analysis (Fig. 8). The expression patterns showed that seven selected genes related to bolting and flowering were differentially expressed in root, stem, leaf and flower of radish (Fig. 8a). Most of them showed a low expression in radish root, but the lowest expression levels of *RsAGL24*, *RsSOC1* and *RsSVP* were in the flower. The relative expression levels of *RsAGL24*, *RsSOC1*, *RsSVP* and *RsVRN2* were significantly higher in the leaf than in the other three tissues, whereas *RsAP2* was mainly expressed in the flower. The transcript levels of *RsFPF1* and *RsLD* were higher in leaf and flower, and no significant difference was detected in expression of these two tissues. The results indicate that these genes may play different roles in various tissues and thus affect the growth and development of radish. The expression pattern differences of these genes were also observed in previous studies [2, 14].

The expression profiling of these seven bolting and flowering-related genes also revealed that their expression patterns in radish leaves varied at vegetative stage and reproductive stage (Fig. 8b), which contributed to further explore their possible roles during radish bolting and flowering. The expression levels of *RsAGL24* and *RsSOC1* were lower at vegetative stage and increased at reproductive stage; whereas the expression levels of these four genes including *RsAP2*, *RsFPF1*, *RsSVP* and *RsVRN2*, were relative higher at vegetative stage and significantly decreased at reproductive stage. The up- or down-expression of these genes were in agreement with previous analysis in other species [24, 25, 51]. Flowering pathway analysis indicated that seven selected genes were implicated in different flowering pathways (Fig. 6). Among them, *AP2* belonging to photoperiod pathway is regulated by miR172 and represses the bolting and flowering [35]; while *SOC1* as a floral integrator negatively regulates *FLC* expression and plays vital roles in promoting flowering [10, 49]. These findings revealed that these differentially expressed genes might participate in the transition from vegetative stage to bolting and flowering in radish.

**Fig. 9:**
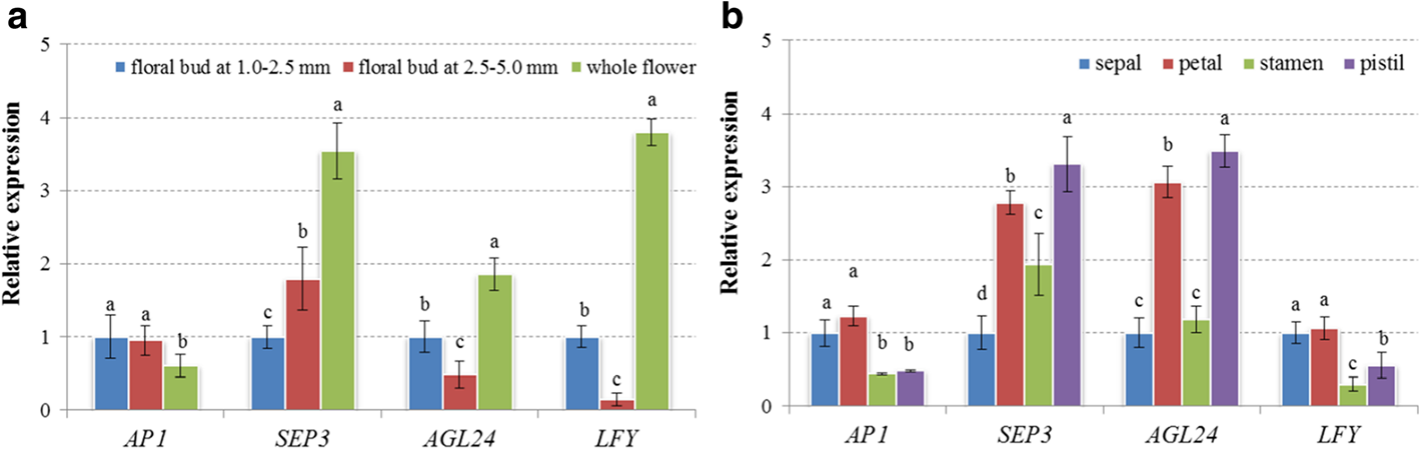
The spatial-temporal expression profiling of flower development-related genes by RT-qPCR analysis. **a** The relative expression levels of four genes at three stages of flower development. **b** The relative expression levels of four genes at different flower parts (sepal, petal, stamen and pistil). Each *bar* shows the mean ± SE of triplicate assays. The values with *different letters* indicate significant differences at *P* < 0.05 according to Duncan’s multiple range tests

Additionally, four flower development-related genes including *RsAP1*, *RsSEP3*, *RsAGL24* and *RsLFY*, were selected for RT-qPCR analysis to explore their expression patterns during radish flower development and in different flower parts. The expression levels of *RsSEP3*, *RsAGL24* and *RsLFY* were relative higher at whole flower, while no significant difference of *RsAP1* expression was showed at three stages of flower development (Fig. 9a). The expression analysis revealed that four genes were differentially expressed in four flower parts (Fig. 9b), which was in consistent with previous studies [51, 56, 58], suggesting that these genes may play important roles in flower organ identity. Importantly, *AP1* belonging to A-class genes in the ABC model of flower organ identity, determines the development of sepal and petal [58, 62]. The results showed that *RsAP1* expression level was relative higher in sepal and petal than in stamen and pistil (Fig. 9b), suggesting that *AP1* gene is essential for sepal and petal development and specify the identity of the floral meristem [62].

## Conclusions

To our knowledge, this is the first report on *de novo* transcriptome sequencing and assembly in late-bolting radish leaves using RNA-seq technology. A total of 53,642 unigenes were assembled in radish leaf transcriptome and 50,385 unique sequences were successfully annotated by GO, COG and KEGG databases. A total of 24 candidate genes were involved in plant circadian rhythmic pathway. Moreover, 142 potential radish genes related to bolting and flowering-time regulation were identified by BLAST similarity search with *A. thaliana* flowering-related genes. RT-qPCR analysis revealed that several flowering-related genes showed spatial-temporal expression patterns in various radish tissues and at different development stages. The findings from this study provided insights into bolting and flowering-time regulatory networks in radish, and facilitated further dissecting molecular genetic mechanisms associated with bolting and flowering regulation in Brassicaceae vegetable crops.

## Methods

### Plant materials

The radish advanced inbred line ‘NAU-LU127’, late bolting and flowering, was used in this study. When cultivated during the autumn season, this late-bolting radish generally bolts until the middle and late of next April. The seeds were sown in plastic pots and cultured in plant growth chamber with 16 h light (25 °C) and 8 h dark (18 °C). The radish leaves were collected at vegetative stage and reproductive stage, respectively. For gene expression validation, the different radish tissues including root, stem, leaf, flower, floral buds at 1.0-2.5 mm and 2.5-5.0 mm, sepal, petal, stamen and pistil, were separately collected at reproductive stage and immediately frozen in liquid nitrogen and stored at -80 °C. All the tissues were harvested with three independent biological replicates.

### Library preparation and Illumina sequencing

Total RNA was isolated using Trizol reagents (Invitrogen, USA) according to the manufacturer’s protocol. For cDNA library construction, a total of 20 μg RNA from radish leaf samples at two different stages were pooled in equimolar quantity. Briefly, the mRNA was isolated using magnetic beads with Oligo (dT), and fragmented to small pieces using fragmentation buffer (Ambion, USA). Then the mRNA fragments were used as templates to synthesize double-stranded cDNA with random hexamer primers using the SuperScript Double-Stranded cDNA Synthesis Kit (Invitrogen, USA). The synthesized cDNA were purified with QiaQuick PCR extraction kit and subjected to end reparation and single nucleotide A (adenine) addition. Thereafter, the short fragments were connected with adapters and the suitable fragments were screened as templates for PCR amplification. The transcriptome library was sequenced using Illumina HiSeq™ 2000 at Beijing Genomics Institute (Shenzhen, China).

### *De novo* assembly of radish leaf transcriptome

The raw reads were initially processed by Illumina sequencing and filtered to produce clean reads by removing adapter sequences, unknown or low quality reads. Transcriptome *de novo* assembly was performed using the Trinity program [36] based on the de Bruijn graph algorithm, with k-mer of 25, minimum k-mer coverage of 1 and minimum contig length of 100. Default settings were used for all other parameters. Clean reads were firstly assembled to form longer fragments named contigs, and consequently the reads with overlapping regions were mapped back to the corresponding contigs. Based on the paired-end reads, the detection of different contigs from the same transcript sequences and the distance among them were detected and calculated, respectively. Then, these contigs were further assembled by the Trinity [36] and the obtained sequences were defined as unigenes with no extension on either end. According to the FPKM method (Fragments Per kb per Million fragments) previously described [37], the expression level of each unigene was calculated with the formula: FPKM = (10^6^ × *C* × 10^3^)/*NL*. Where C is the number of reads that uniquely aligned to certain unigene, N is the total number of reads that uniquely aligned to all unigenes, and L is the number of bases on this unigene.

### Functional annotation and classification

All the assembly unigenes were searched and annotated against the publicly available protein databases including NR, Swiss-Prot, KEGG and COG, using BLASTx analysis with an *E*-value cut-off of 1.0E-05. Based on the BLAST results, the CDS of unigenes were predicted for proteins by using the best alignments, which conducted following a priority order of NR, Swiss-Prot, KEGG and COG. If a unigene could not be aligned to these databases, ESTScan software was used to decide the sequence direction [63], including the nucleotide (5'-3') and amino acid sequences of the coding regions. Based on NR annotations, the Blast2GO program was employed to gain GO annotations of unique transcripts at the second level, according to biological processes, cellular components and molecular functions [64]. GO functional classification and distribution of gene functions of each assembly unigene were performed using WEGO software at the macroscopic level [65].

### Identification and isolation of bolting and flowering- related genes

According to the reported flowering regulatory network in *A. thaliana* [2, 3], the complete cDNA sequences of flowering-related genes were downloaded from the public NCBI database. The assembly transcript sequences from ‘NAU-LB’ were subjected to a local BLASTx similarity search at *E*-value ≤ 1.0E-05 against the *A. thaliana* gene sequences. The phylogenetic analysis was performed using the MEGA 6.0 software (http://www.megasoftware.net/) with the Neighbor-Joining algorithm. The sequences of *SOC1* homologs used for phylogenetic tree construction were retrieved from the NCBI database. For T-A cloning, first-strand cDNA was synthesized from total RNA in radish leaves with the M-MLV (RNase H^-^) reverse transcriptase (TaKaRa, Dalian, China). Specific PCR primers were designed using Primer Premier 5.0 and shown in Additional file 6. The PCR amplification was performed according to previously reported method [32]. Purified PCR products were ligated into the pMD18-T vector (TaKaRa, Beijing, China), and then transformed into *E. coli* DH5α. Positive clones were sequenced on an ABI3730 sequencer (Applied Biosystem, USA).

### RT-qPCR analysis

RT-qPCR analysis was performed to validate the relative expression levels of several flowering genes in diverse radish tissues and at different stages. Total RNA from various tissue samples was isolated and reverse transcribed into cDNA as described above following the manufacturer’s instructions, respectively. The specific primers for RT-qPCR were designed using Beacon Designer 7.0 (Premier Biosoft International, USA). Three biological replicates were performed for RT-qPCR assay to ensure the reliability of quantitative analysis. The PCR reaction was conducted using an iCycler Real-Time PCR Detection System (Bio-Rad, USA) and the *RsActin* gene was used as the reference gene [66]. The relative fold expression changes were calculated using the 2^-∆∆*C*T^ method [67]. The error bars representing the standard deviation were derived from each sample in triplicate. The statistical analysis with SAS Version 9.0 software (SAS Institute, Cary, North Carolina, USA) was performed using Duncan’s multiple range test at the *P* < 0.05 level of significance. All primer pairs used in this study are listed in Additional file 6.

## Additional files

Additional file 1: Summary of all the assembled unigenes in radish leaf transcriptome. (XLSX 14582 kb) Additional file 2: KEGG pathways of the assembled unigenes in radish. (XLSX 130 kb) Additional file 3: Candidate genes involved in circadian rhythm of radish. (XLSX 107 kb) Additional file 4: Identification of flowering related genes in radish. (XLSX 57 kb) Additional file 5: Annotation of transcript sequences related to flowering genes in radish. (XLSX 139 kb) Additional file 6: Specific primer sequences of flowering related genes for T-A cloning and RT-qPCR. (XLSX 11 kb)

## Abbreviations

*CO*: *CONSTANS*
COG: Clusters of Orthologous Groups
*FLC*: *FLOWERING LOCUS C*
FPKM: Fragments Per kb per million fragments
*FT*: *FLOWERING LOCUS T*
GO: Gene ontology
KEGG: Kyoto Encyclopedia of Genes and Genomes
*LFY*: *LEAFY*
NGS: Next-generation sequencing technology
ORF: Open reading frame
RNA-seq: RNA sequencing
RT-qPCR: Reverse transcription quantitative real-time PCR
*SOC1*: *SUPPRESSOR OF OVEREXPRESSION OF CONSTANS 1*
